# Advancing the Characterization of Recycled Polyolefin Blends with a Combined Experimental and Numerical Approach to Thermomechanical Behavior

**DOI:** 10.3390/polym16081153

**Published:** 2024-04-19

**Authors:** Pei Hao, Charmayne Siebers, Kim Ragaert, Francisco A. Gilabert

**Affiliations:** 1 Department of Materials, Textiles and Chemical Engineering (MaTCh), Mechanics of Materials and Structures (MMS), Tech Lane Ghent Science Park-Campus A, Ghent University (UGent), Technologiepark-Zwijnaarde 46, 9052 Ghent, Belgium; pei.hao@ugent.be; 2 Circular Plastics, Department of Circular Chemical Engineering, Faculty of Science and Engineering, Maastricht University, Urmonderbaan 22, 6167 RD Geleen, The Netherlands; charmayne.siebers@maastrichtuniversity.nl (C.S.); k.ragaert@maastrichtuniversity.nl (K.R.)

**Keywords:** polyethylene, polypropylene, recycled plastics, mechanical properties, self-heating, finite element analysis

## Abstract

The blending of polyolefins (POs), such as polyethylene (PE) and polypropylene (PP), is a growing area of research, particularly for recycling mixed polyolefin (MPO) waste through flotation sorting techniques. However, understanding the thermomechanical behavior of these recycled blends is challenging due to limitations in the existing characterization methods. This paper introduces a combined experimental and numerical method to accurately assess the complex mechanical behavior of high-density PE, PP, and their blends. We conducted detailed thermomechanical analyses using a high-speed stereo digital image correlation (DIC) system paired with an infrared camera to capture temperature variations alongside mechanical stress and strain. This approach allowed us to correct for distortions caused by necking and to derive accurate stress–strain relationships. We also applied a cutting-edge unified semi-crystalline polymer (USCP) model to simplify the analysis, focusing on the effects of strain rate and temperature, including self-heating and thermal softening phenomena. Our results, which closely match experimental observations of stress–strain behavior and temperature changes, offer new insights into the thermomechanical properties of PO blends, which are essential for advancing their practical applications in various fields.

## 1. Introduction

Plastics now play an indispensable role in our everyday routines, serving diverse purposes across various applications, including packaging, household products, construction, electronics, healthcare, and the automotive industry, owing to their light weight, low cost, easy processability, and tunable properties [1]. They are the largest synthetic consumer product in the world, with an annual production reaching 400.3 million tonnes (Mt) globally in 2022 [2]. Research and innovation for dealing with plastic waste is imperative to advance a circular economic framework [3]. Among all the plastic wastes, two dominant types of thermoplastics are polyethylene (PE) and polypropylene (PP), constituting over 30% [2]. Typically, there exist four prevalent types of PE: ultra-high-molecular-weight polyethylene (UHMWPE), high-density polyethylene (HDPE), low-density polyethylene (LDPE), and linear low-density polyethylene (LLDPE). As the main packaging plastics, these polyolefins can be recycled mechanically thanks to their ability to be reshaped [4], and this circular solution is foreseen to keep dominating by 2050 [5].

However, the current mechanical recycling (MR) approaches do not enable the attainment of high-purity recyclates from a single polymer type due to the complex and challenging nature of the plastic waste stream [6,7]. A polymer blend is defined as a mixture of at least two macromolecular substances, polymers or copolymers, in which the content of ingredients is above 2 wt% [8]. The flotation-based sorting procedure leads to polymer blends inevitably comprising various polymers, owing to their similar properties such as melting temperatures and densities. Polyolefin blending has been an active area of research for several years considering the mixed polyolefin (MPO) waste fraction separated using current sorting techniques [9,10,11,12]. On top of mis-sorted polymers, additives, contaminants, and multilayer products can be found in the plastic waste stream, resulting in substantial property deterioration during and after reprocessing. Therefore, MR post-consumer plastics frequently produces downcycled materials with lower quality and/or utility [1], causing their low acceptance in secondary markets.

For recycled MPO materials to re-enter the consumer market, the composition quality and thermomechanical performance of recycled blends must be evaluated. In general, a testing campaign following standard mechanical tests is the most adopted method. Pioneering work has been conducted to investigate the effect of in situ reactive compatibilizers on the improvement of PP/LDPE blends in terms of tensile strength, elongation at break, and impact strength [13]. Wu and Wang [14] experimentally explored different compositions of PP/PE blends and found that the combination 10%PP/30%LLDPE/60%HDPE achieves the best toughening, impact strength, and failure strain compared to pure HDPE. Van Belle et al. [15] performed a comprehensive experimental study on different binary blending systems using structurally similar polyolefins and correlated the changes in mechanical properties to the deformation mechanisms. Later, Demets et al. [16] adopted a similar strategy to construct the structure–property relationships for four dominant types of POs under tensile loads, but contaminated with three different non-polyolefin (NPO) polymers. More recently, Jones et al. [17] examined the variability in the thermomechanical behavior of virgin and recycled PP/HDPE blends without the addition of other components. The tensile properties of recycled blends were found to be inferior to those of virgin blends due to the deterioration during the recycling process. Gavande et al. [18] blended a novel ultra-high-molecular-weight polypropylene (UHMWPP) to HDPE and investigated the effects of UHMWPP on the mechanical, thermal, and rheological properties. Certainly, the experimental tests can provide an indication of the performance of polymer blends, but the research–development loop becomes excessively costly and time-consuming when considering various loading cases.

A powerful, straightforward and robust tool towards predictive design is still highly required to assess the performance of recycled plastics. Thanks to the toughening mechanism, polycarbonate (PC)/acrylonitrile-butadiene-styrene (ABS) polymer blends are the most studied material using different numerical analyses. A microscale-based numerical study on PC/ABS polymer blends was conducted to investigate the dependence of blend composition and phase properties [19]. The heterogeneous material, ABS, was assumed by a homogenized material to correlate the toughening mechanisms to the blend microstructure. This material model was also applied to study the crack tip plasticity in polymer/rubber blends [20]. Alternatively, a phenomenological constitutive model has been constructed for predicting a wide range of temperatures and high-strain-rate behaviors of PC/ABS blends [21]. Based on a DSGZ model, a modified phenomenological constitutive model was proposed to account for the loading and unloading [22]. However, the modeling work on MPO consisting of structurally similar phases is significantly limited. Drozdov et al. [23] developed a model for the viscoelastic and viscoplastic responses of PP/PE blends with arbitrary three-dimensional deformation with small strains. Due to the fact that both PP and PE are classified as semi-crystalline polymers (SCPs), the effect of annealing was studied by decomposing the inelastic response PP/PE blend into two phases: amorphous and crystalline ones [24]. In practice, a homogenization concept is applied to binary blends of SCPs with a complicated microstructure. The mixture is represented as a unified single-phase continuum, exhibiting mechanical properties aligned with those of the blend.

It is important to remark that polymer blends are not the only multi-phase materials, but pure thermoplastics also are. Most thermoplastics are classified as SCPs, combining amorphous and crystalline phases [25,26,27,28]. A recent developed mesoscopic-based model provides a robust identifiable relationship between the amorphous–crystalline phase interaction and the overall stress–strain response [29]. It is widely recognized that polymers are pressure-, rate- and temperature-dependent, and they also suffer from self-heating and thermal softening effects [30,31,32,33]. To assess the intrinsic thermomechanical response, efforts have been made using digital image correlation (DIC) for video-monitored testing [32,33,34]. Poulain et al. [34] showed that different video-based extensometry techniques lead to significant differences in the stress–strain responses of ductile polymers under large strains. With the growing usage of recycled plastics by end-users, it becomes imperative to thoroughly investigate the intrinsic thermomechanical response of polymer blends.

In this paper, a state-of-the-art experimental investigation was conducted to retrieve the thermomechanical response of polyolefins and their blends under tension at various loading speeds. The numerical study was performed using an advanced polymer model and extended its application to (i) pure SCPs above the glass transition temperature and (ii) polymer blends. Section 2 presents the materials, compounding process, sample preparation, and detailed mechanical testing process to ensure the acquisition of intrinsic material response. Section 3 describes the constitutive equations of the polymer model and the application to pure polymers and blends within a thermomechanical coupling framework. The model is calibrated, and material parameters are provided for the model validation and prediction at different loading speeds. Section 4 is devoted to applying the advanced polymer model to the characterized polymers under tension. This section presents the model prediction of pure polymers and their blends, showing different types of trends in stress–strain curves, incorporating the self-heating effects.

## 2. Experimental Methodology

This section describes the procedures for sample preparation, the setup for tensile testing, and the post-processing of data. The process of data acquisition utilizing 3D digital image correlation (DIC) and the synchronization of input sources from infrared (IR) cameras are elucidated. The full-field strain and temperature measurements enable access to the intrinsic thermomechanical coupled response.

### 2.1. Materials and Compounding Process

In this study, blow molding and injection molding grades of HDPE and PP (F4520 and 576P) were provided by SABIC^®^, Geleen, The Netherlands. SABIC^®^ 576P is an isotactic PP homopolymer grade known for its good flow properties and narrow molecular weight distribution; it is typically used in caps, closures, and thin-wall packaging. The specific grade, SABIC^®^ F4520, is suitable for blow molding packaging applications. A blend containing 10 wt% 576P was created using a Collin co-rotating twin screw extruder, model TEACH-LINE ZK 25T, with a screw diameter of 25 mm and an L/D ratio of 18. The temperature profile ranged from 180 °C to 200 °C at 140 rpm. Due to the intense shearing forces and energy input from the screws, the actual material temperature at the nozzle was measured around 220 °C. The pure HDPE and PP samples were also subjected to melt processing under the same conditions. The strand emerging from the die was submerged into a cooling water tank and finally chopped into pallets 3–4 mm in size.

### 2.2. Injection Molding and Specimen Preparation

The tensile test specimens (ISO 527-2:2012, type-1A [35]) were molded on a Boy E35E injection molding machine with a 28 mm screw diameter and an L/D ratio of 18.6. The temperature profile from the hopper to the nozzle was set between 185 and 210 °C. The mold temperature was maintained at 40 °C for all materials. The injection molding process settings are listed in Table 1.

**Figure 1:**
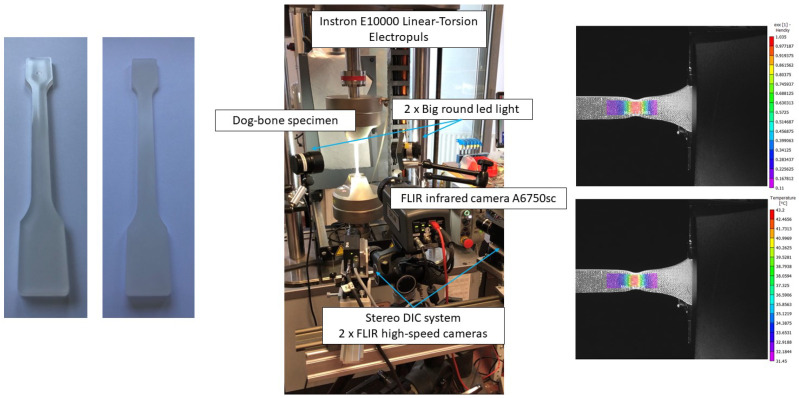
ISO-527 type-1A dog-bone specimen preparation (**left**), experimental setup (**middle**), and DIC measurements (**right**).

To achieve the flat surface shown in Figure 1 (left), the dog-bone samples were polished using disc grit up to 1200 on a Struers LaboPol-60 equipped with a water cooling system. This facilitated the application of a DIC speckle pattern using a speckle stamps kit set and avoided stress concentration during the tensile test. An acrylic, water-based ink with a mat white background was applied using an airbrush gun technique, resulting in a homogenous substrate layer without peeling off from the specimen surface due to large strains. To produce the most accurate results, a random speckle pattern was applied to the specimen’s surface using speckle stamps from Correlated Solutions, Inc. The speckle quality was strictly examined to ensure it was neither too sparse nor too dense, in order to yield consistent results and minimize noise levels.

### 2.3. Tensile Tests

Experiments were conducted following ISO 527-1:2012 [36] using an electromechanical Instron ElectroPuls E10000 tensile apparatus equipped with a 10 kN static load cell and pneumatic grips. The pressure was set at 2.0 bar. Figure 1 (middle) presents the experimental setup that equips two monochrome cameras, an infrared (IR) camera, and data acquisition system. Using IR camera inspection, it records the temperature evolution in the specimens and correlates to the stress–strain curve. Loading speeds of the cross-head were chosen from 1 to 40 mm/min to cover the isothermal, thermal-coupled, and nearly adiabatic scenarios, respectively. All tests were conducted at room temperature (RT). A LabVIEW code and corresponding hardware integration were developed to trigger three cameras and to acquire the force and displacement signal exported from the test bench in a synchronized manner. Images for the stereo-DIC analysis and IR camera were acquired at adjustable frequencies ensuring 2000 frames during a single tensile test. The load cell was calibrated to produce a 10 V output at 10 kN, using a 10 kN Instron quasi-static load cell for in-series calibration.

### 2.4. Temperature Field Mapping and True Strain–Stress Response

**Figure 2:**
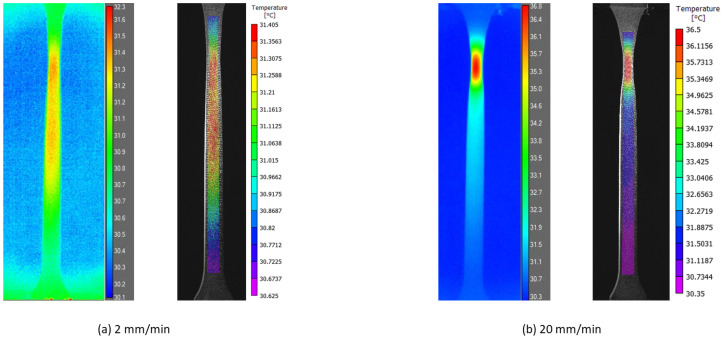
Temperature field mapping from IR camera recording to DIC post-processing of PP/HDPE blend at different loading speeds.

Two monochrome cameras, GS3-U3-51S5M, equipped with lens Kowa, LM35JC were built for the 3D stereo-DIC system. The high resolution of 2448 × 2048 pixels and fast data acquisition using USB 3.0 enabled us to capture sufficient frames during a short period event, such as 40 mm/min. An FLIR A6750sc IR camera was synchronized to record the temperature field on the specimen surface through a common trigger box. The 3D stereo-DIC system was calibrated using the commercial software VIC3D (version 9.4.22). The stereo-DIC setup had a stand-off distance of approximately 731 mm, a stereo angle of around 22.45°, and a magnification of about 13.5 pixels per millimeter in the images. Temperature field recorded using FLIR ResearchIR software (version 4.40.12.38) was then imported as external camera data in VIC3D post-processing. Figure 2 shows the mapping results of temperature field synchronized with DIC images. The PP/HDPE blend tested at two different loading speeds is used to illustrate the thermomechanical coupling effect under isothermal and thermal coupled scenarios. The synchronization of strain and temperature measures facilitates the quantitative analysis of self-heating and thermal softening effects.

To obtain the average axial Hencky strain, the subset and step sizes were selected as 29 and 8 pixels to establish correlation among the speckles, which corresponded to 2.15 and 0.59 mm, respectively. The true stress estimation was calculated considering the yield drop resulting from the necking instability of plastic materials [34]. As incompressibility (i.e., $A_{0}/A=L/L_{0}$) and isotropy are assumed in the plastic regime, the true stress can be approximately obtained:

(1) $$\sigma^{\mathrm{app}}=\frac{F}{A_{0}}\exp\left(\varepsilon \right)$$

where *F* is the applied force, $A_{0}$ is the initial area of the cross-section, and ε is the average Hencky strain along the loading direction.

**Figure 3:**
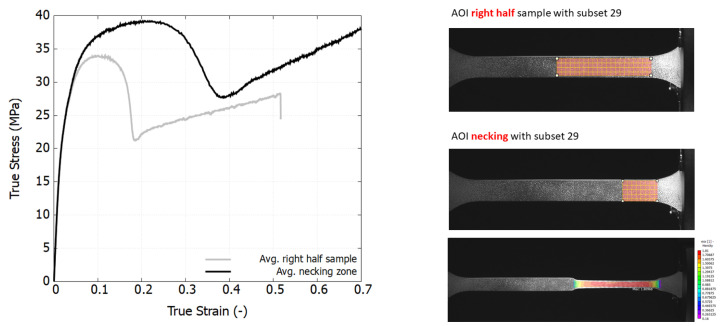
The effect of the Area of Interest (AOI) on true stress–strain response of a PP 576P specimen at a loading speed of 10 mm/min.

However, decoupling the intrinsic material behavior from the necking effect was a challenge. As shown in Figure 3, necking does not occur across the entire specimen gauge area, leading to non-homogeneous deformation, resulting in shear yielding and highly stretched regions. This phenomenon primarily originates from the combined effects of geometrical defects and material inhomogeneity generated from the injection process (i.e., density and stiffness). To illustrate different descriptions of the post-yield response depending on the strain extraction, two representative Areas of Interest (AOIs) were chosen from the PP specimen and underwent a loading speed 10 mm/min. The so-called true stress–strain curve in grey corresponds to the AOI from half of the sample with the area approximated to 8.6 × 45.15 mm^2^, whereas the black one was determined according to the necking zone at the last frame of the test (close to 9.67 × 16.12 mm^2^). Because the deformation within the non-necking zone does not progress during the plastic regime, the curve obtained from the necking zone was used in this study.

## 3. Thermomechanical Modeling

This section outlines the key features of the constitutive polymer model that was recently developed and validated for various SCPs. A detailed description of the Unified SCP (USCP) model, including full mathematical formula derivation, can be found in Reference [29]. The USCP model was developed in response to the observation of a double yield phenomenon in SCPs, as documented in References [37,38,39]. In this study, the USCP model was applied directly to pure HDPE, PP, and the PP/HDPE blend. The thermal and mechanical properties of this binary blend are considered as those of a unified single continuum with an SCP structure.

### 3.1. Constitutive Model

**Figure 4:**
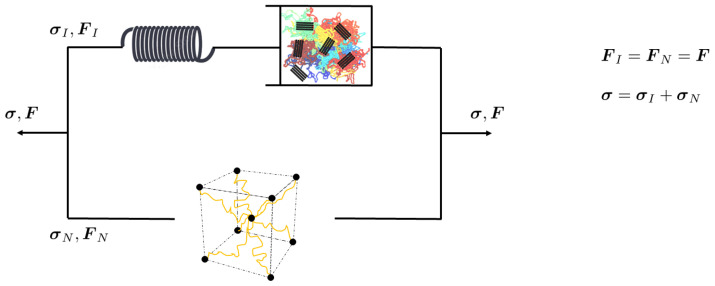
Schematic representation of intermolecular and network resistances in corresponding rheological model.

This model, formulated within the finite strain kinematic framework, is a generalization of the Boyce–Parks–Argon (BPA) model [40] by incorporating a single viscoplastic law that unifies the amorphous and crystalline phases of the polymer. Figure 4 illustrates the rheological analogue and the corresponding behavior.

The Cauchy stress tensor in the intermolecular branch $\sigma_{I}$ is obtained by eliminating the plastic deformation gradient $F_{A}^{p}$.

The rate of inelastic deformation is written as

(2) $${\tilde{D}}_{I}^{p}=\dot{\bar{\varepsilon }}\;N,$$

where N is the direction tensor and $\dot{\bar{\varepsilon }}$ is the effective plastic strain rate.

The evolution law for the athermal effective stress is formulated using a smooth, heaviside-like function to characterize the pre-peak hardening, post-peak softening, and second yield due to the crystalline phase contribution, as follows:

(3) $$\dot{s}=H_{1}\left(\bar{\varepsilon }\right)\cdot \left(1-\frac{s}{s_{1}}\right)\cdot \dot{\bar{\varepsilon }}+H_{2}\left(\bar{\varepsilon }\right)\cdot \left(1-\frac{s}{s_{2}}\right)\cdot \dot{\bar{\varepsilon }}+H_{3}\left(\bar{\varepsilon }\right)\cdot \left(1-\frac{s}{s_{3}}\right)\cdot \dot{\bar{\varepsilon }},$$

where athermal strength $s_{i}$ (i = 1,2,3) correspond to the preferred state at different stages [29]. It involves three hardening (softening) parameters, $h_{1}$, $h_{2}$, and $h_{3}$; the smoothing factor, *f*; plastic strain at the peak yield; and a typical peak yield along with two saturated states representing athermal strength ($s_{1}$, $s_{2}$, and $s_{3}$). The functions controlling the hardening evolution are given by

(4) $$H_{1}\left(\bar{\varepsilon }\right)=-h_{1}\left\{\tanh\left(\frac{\bar{\varepsilon }-{\bar{\varepsilon }}_{p}}{f{\bar{\varepsilon }}_{p}}\right)-1\right\},$$

(5) $$H_{2}\left(\bar{\varepsilon }\right)=h_{2}\left\{-\tanh\left(\frac{\bar{\varepsilon }-{\bar{\varepsilon }}_{p}}{f{\bar{\varepsilon }}_{p}}\right)\tanh\left(\frac{\bar{\varepsilon }-{\bar{\varepsilon }}_{c}}{f{\bar{\varepsilon }}_{c}}\right)+1\right\},$$

(6) $$H_{3}\left(\bar{\varepsilon }\right)=h_{3}\left\{\tanh\left(\frac{\bar{\varepsilon }-{\bar{\varepsilon }}_{c}}{f{\bar{\varepsilon }}_{c}}\right)+1\right\},$$

where ${\bar{\varepsilon }}_{p}$ is the plastic strain at the peak yielding point and ${\bar{\varepsilon }}_{c}$ is the characteristic plastic strain when the crystalline nano-block initiates the yielding process.

The stress contribution of the network resistance $\sigma_{N}$ depends on the rubbery modulus $C^{R}$ and the number of rigid links *N*, namely

(7) $$\sigma_{N,i}=\frac{1}{3}C^{R}\sqrt{N}\frac{\lambda_{i}^{p2}-\lambda^{p2}}{\lambda^{p}}L^{-1}\left(\frac{\lambda^{p}}{\sqrt{N}}\right)$$

where $\lambda_{i}^{p}$ is the plastic stretch on each chain in the network, $\lambda^{p}$ is the root-mean-square of the applied plastic stretches, the symbol $L^{-1}$ is the inverse Langevin function, and $C^{R}$ and *N* are the rubbery modulus and the average number of links between entanglements, respectively.

### 3.2. Thermomechanical Coupling

All materials investigated in this study exhibited self-heating and thermal softening effects, as demonstrated by the experimental results. To quantitatively assess the temperature increase resulting from self-heating, understanding thermomechanical coupling is essential. The deformation-related plastic dissipation must be accounted for in the heat balance equation:

(8) $$\rho \;c_{p}\frac{\partial \theta }{\partial t}=\eta \sigma_{I}:{F^{e}}_{I}{{L^{p}}_{I}\left({F^{e}}_{I}\right)}^{-1}+\nabla \cdot \left(k\frac{\partial \theta }{\partial x}x\right),$$

where ρ is the density of the polymer. The second-order tensor ${F^{e}}_{I}$ is the elastic deformation gradient on the branch I. The plastic velocity gradient expressed in the relaxed configuration is denoted by ${L^{p}}_{I}$. In all cases, the dissipation is assumed to be completely converted to heat (i.e., η = 1). The thermal specific heat, $c_{p}$, and thermal conductivity, *k*, are assumed to be constant throughout this analysis. The temperature increase is attributed to the interplay between (a) the energy dissipated through plastic deformation and (b) the thermal properties of the material. The energy dissipated as plastic deformation was converted into heat, determining the maximum temperature achievable under adiabatic conditions (characterized by very high loading speeds). Conversely, the thermal diffusivity of the material influenced how the temperature decreased over the testing period, as described by the heat balance equation.

**Figure 5:**
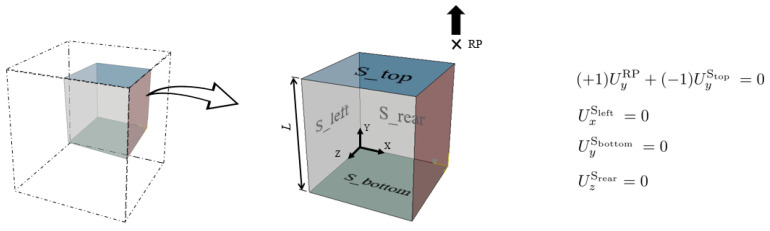
A one-eighth finite element (FE) model with corresponding boundary conditions.

The model was implemented in Abaqus by incorporating both user subroutines UMAT and UMATHT. The volumetric heat generation per unit time at the end of each increment, attributable to the material’s own plastic dissipation, is calculated as specified in Equation (8). A single element (SE) test was developed to iteratively perform the model calibration procedure using the Nelder–Mead optimization method. The element type, C3D8RT, characterized by eight-node trilinear displacement and temperature, employed reduced integration with hourglass control. Figure 5 depicts the boundary conditions (BC) applied to a one-eighth cube geometrical symmetry. The top surface is linked to a reference point (RP) where a displacement controlled is applied. Initially, a predefined temperature field was set for the entire model. Structural symmetry was considered for the left, rear, and bottom surfaces by restricting corresponding degrees of freedom. A film coefficient *h*, representing heat conduction to the ambient temperature, was applied to all free surfaces to simulate heat transfer from the surrounding continuum to this material point.

### 3.3. Model Parameters: Mechanical and Thermal

A two-step parameter identification (PI) procedure using a (i) standard PI and (ii) SE test was employed to calibrate the material model [32]. The constrained Nelder–Mead optimization automatically retrieved the parameters $s_{3}$, $h_{3}$, and ${\bar{\varepsilon }}_{c}$ by minimizing the difference between the experimental result and the simulation one. The model fundamentally requires two stress–strain curves at different loading speeds (or strain rates) for effective characterization. In this study, PP was specifically identified as our primary pollutant of interest, and we pursued a strategy aimed at minimizing experimental calibration efforts for efficient industrial application. Readers are referred to the calibrated parameters for the USCP model in Table 2 with the corresponding thermal properties for the investigated polymers used in this study.

Thermal properties also have an important effect on the stress–strain curves owing to the fact that thermal softening relies on the actual temperature. According to the heat balance equation (see Equation (8)), density ρ, specific heat $c_{p}$, and thermal conductivity *k* must be provided. For the PP/HDPE blend, the empirical Neumann–Kopp law was adopted to provide the estimation of the density [41,42] and the heat capacity [43,44] for a mixed material, which are given by

(9) $$\rho_{\mathrm{blend}\;}=\sum_{i=1}^{i=n}\chi_{i}\rho_{i},\;c_{p,\mathrm{blend}\;}=\sum_{i=1}^{i=n}\chi_{i}c_{p,i}$$

where $\chi_{i}$ is the weight fraction of each blending component.

The thermal conductivity of the blend was calculated using the classical Maxwell–Eucken model, as follows:

(10) $$k_{\mathrm{blend}}=k_{\mathrm{HDPE}}\frac{2k_{\mathrm{HDPE}}+k_{\mathrm{PP}}+2V_{f,\;\mathrm{PP}}\left(k_{\mathrm{PP}}-k_{\mathrm{HDPE}}\right)}{2k_{\mathrm{HDPE}}+k_{\mathrm{PP}}-V_{f,\;\mathrm{PP}}\left(k_{\mathrm{PP}}-k_{\mathrm{HDPE}}\right)}$$

where $V_{f,\;\mathrm{PP}}$ is the volume fraction of the PP phase.

## 4. Results and Discussion

### 4.1. Experimental Stress–Strain Response

**Figure 6:**
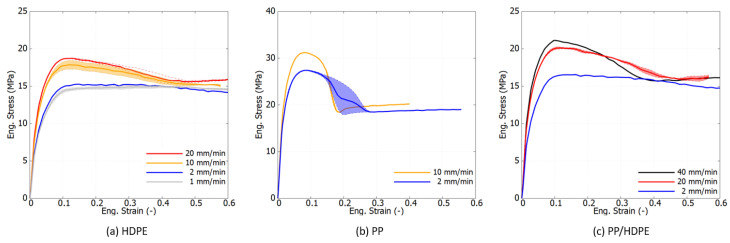
Experimental engineering stress–strain curves under tension at different strain rates at room temperature.

Figure 6 presents the averaged engineering stress–strain curves, along with the upper and lower bounds depicted in shadow from repeated experiments. The engineering measurements do not isolate the effects of geometric nonlinearity from the dog-bone specimen. Engineering stress is determined directly from the force output of the tensile apparatus without accounting for the shrinkage of the specimen caused by the necking process.

Engineering stress–strain curves are widely recognized in industrial applications as an efficient and standardized method for evaluating products and generating datasheets. As depicted in Figure 6, the three materials tested exhibit distinct stages during tensile deformation: elastic, viscoplastic, peak yield, necking initiation, and necking stabilization. Notably, the stress–strain response of PP is significantly different compared to the HDPE and PP/HDPE blends, exhibiting a higher peak yield stress of 32 MPa and an earlier stage of necking stabilization, characterized by a stress plateau during cold drawing. The stress–strain response of the PP/HDPE blend closely resembles that of HDPE, due to the smaller weight fraction of PP (10 wt%) in the blend. Both pure polymers and the blend demonstrate a high rate of dependence. The peak yield stress of HDPE increases from 15 to 18 MPa with an increase in loading speed from 2 to 20 mm/min, a trend also observed in PP and the PP/HDPE blend.

**Figure 7:**
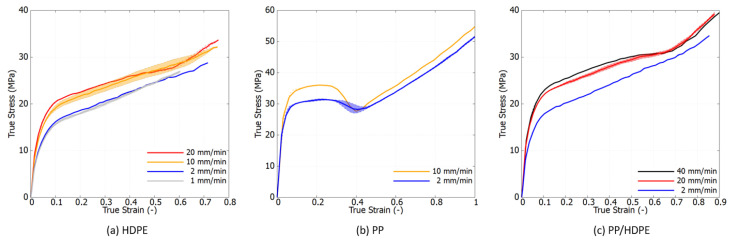
Experimental true stress–strain curves under tension at different strain rates at room temperature.

From a scientific perspective, it is crucial to characterize materials based on their intrinsic response by eliminating the effects of necking. Figure 7 displays the true stress–strain curves, derived using the Hencky strain extraction and true stress approximation methods described in Section 2. Within the examined loading speed range, the viscoelastic response of both polymers is minimally observed. In contrast to the engineering stress–strain curves, the true stress–strain curves exhibit a marked change in trend, revealing a rapid stress increase at large strain levels. This hyperelastic response results from the rubbery effect, which causes reorientation of the polymer chains and predominates the material behavior, as documented in [40,45]. Notably, in the strain range of 30% to 60%, the stress–strain curves at high strain rates show a declining trend that converges towards that at a lower strain rate. This phenomenon is attributed to thermal softening, as polymers are thermally sensitive. Under high-speed loading, self-heating occurs, but the brief duration is insufficient to dissipate the heat, leading to the degradation of material properties at large strains.

### 4.2. Self-Heating Production and Thermal Softening

**Figure 8:**
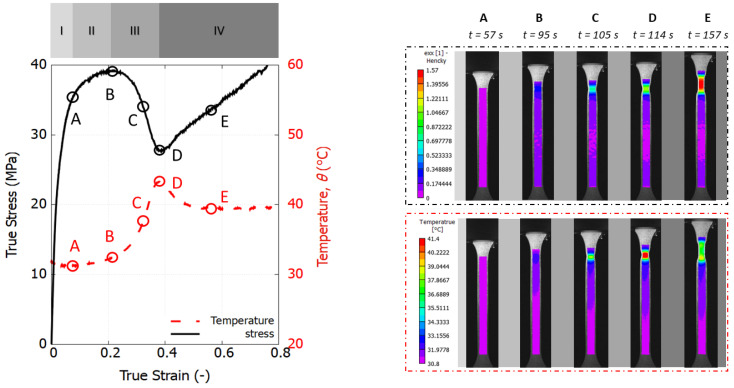
Experimental stress–strain response and temperature evolution of a PP 576P specimen under tension at 10 mm/min (**left**); local profile of strain and temperature fields (**right**).

To further investigate the self-heating effects, PP tested at the loading speed of 10 mm/min was chosen due to its larger plastic dissipative energy, resulting in a more pronounced temperature rise. Figure 8 displays four stages captured throughout the whole tensile deformation of the dog-bone specimen, marked by five investigative instances from A to E. Additionally, the evolution of peak temperature on the specimen surface is plotted alongside the true stress–strain curve.

The initial stage, up to point A, is the elastic regime, during which the temperature slightly decreases from 31.8 to 31.2 °C. This reduction in temperature is indicative of the thermoelastic effect, which occurs when an elastic material is stretched. As the strain increases, Stage II (from instance A to B) is marked by a slight increase in temperature, signaling the onset of plastic deformation. The strain field remains homogeneous up to this point, reaching a peak yield stress of 39.3 MPa. Beyond instance B, necking begins, accompanied by a rapid increase in temperature until it reaches a turning point at D. The maximum temperature observed during the tensile test is 43 °C, occurring as a distinct necking zone forms and heat concentrates in the central zone. In Stage IV, exemplified by instance E, the necking zone undergoes significant stretching. The high stress resistance prevents further deformation and there is not more plastic dissipation within the necking zone. Therefore, the temperature experiences a drop caused by natural convection leading to the stabilization of the necking.

### 4.3. Results Comparison between Experiments and Simulations

**Figure 9:**
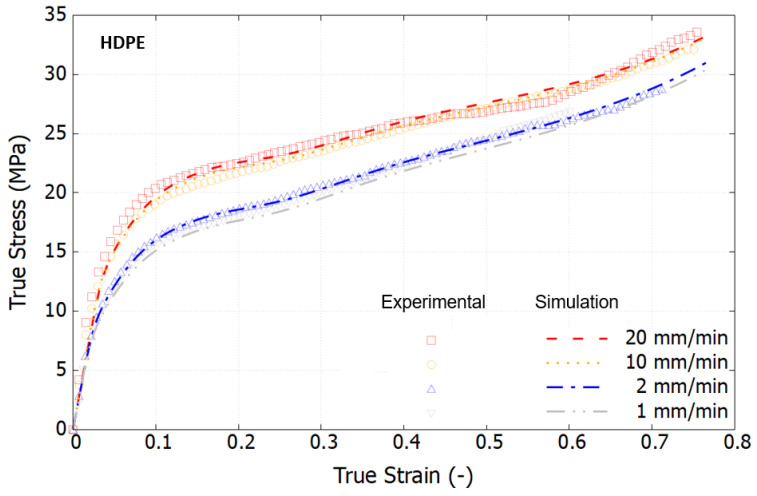
Comparison of the true stress–strain curves of HDPE between the model and the test at different loading speeds.

**Figure 10:**
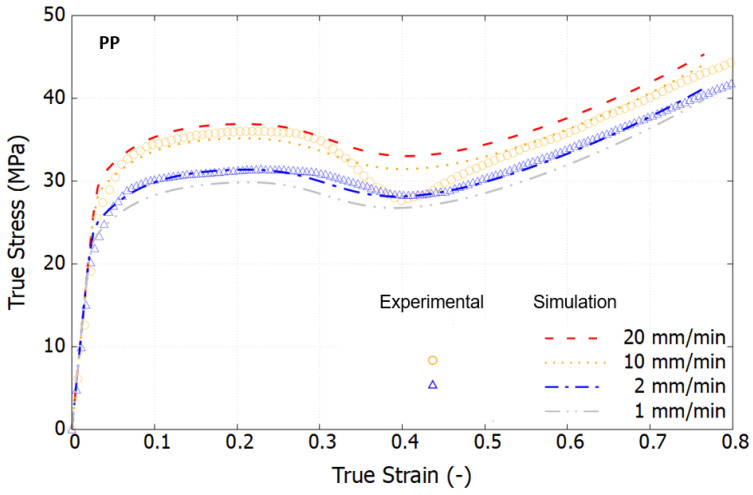
Comparison of the true stress–strain curves of PP between the model and the test at different loading speeds.

**Figure 11:**
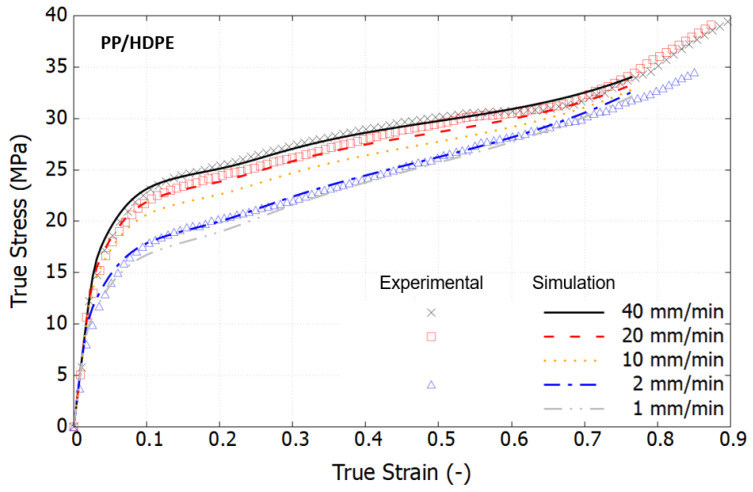
Comparison of the true stress–strain curves of the PP/HDPE blend between the model and the test at different loading speeds.

Figure 9, Figure 10 and Figure 11 compare the simulation results, which incorporate full thermomechanical coupling, with the experimental outcomes. The comparisons show good agreement, capturing several key features of the three investigated materials: HDPE, PP, and the PP/HDPE blend.

In the case of HDPE, the identification process was carried out at a low loading speed of 2 mm/min, and the results align closely with the experimental data. The self-heating and thermal softening effects are distinctly observed in the experimental true stress–strain curves, and the simulations accurately replicate these phenomena.

For PP, the true stress–strain curves exhibit a stress drop preceding the transition into hyperelastic behavior. The advanced constitutive polymer model, designed with a specific evolution of athermal strength, adeptly captures this characteristic trend. The PP/HDPE blend demonstrates higher stress levels in comparison to pure HDPE, which are attributable to the reinforcing effect of PP. Specifically, the initial peak yield stress at a strain of 13% escalates from 17.1 to 18.8 MPa at a loading speed of 2 mm/min, and from 21.2 to 23.1 MPa at a loading speed of 10 mm/min. The sensitivity to the loading rate exhibits notable similarity between HDPE and the PP/HDPE blend.

### 4.4. Temperature Evolution Due to Mechanical Self-Heating

**Figure 12:**
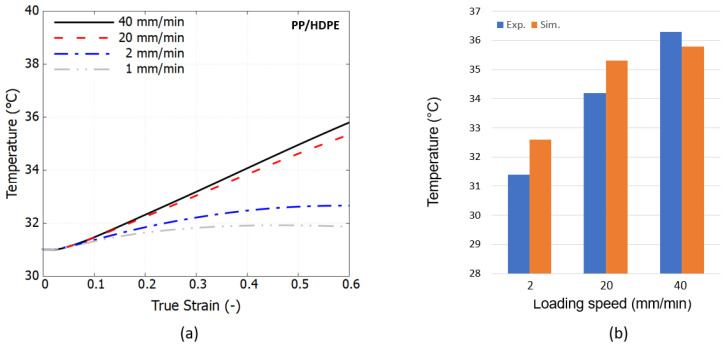
Temperature evolution: (**a**) prediction at different loading speeds using SE test and (**b**) comparison to experimental measured peak temperature at true strain of 60%.

This section shows the predicted temperature evolution versus the true strain of the PP/HDPE blend at four different loading speeds. Figure 12a shows that the temperature increases monotonically and saturates for lower speeds 1 and 2 mm/min beyond the strain level of 40%.

The comparison of the experimental and simulation results reveals consistent trends concerning the effects of loading speed, as illustrated in Figure 12b. With the increase in loading speed from 2 to 20 to 40 mm/min, the peak temperature measured in the experiments rises from 31.4 to 34.2 to 36.3 °C, respectively, whereas the simulations yield temperatures from 32.6 to 35.3 to 35.8 °C, respectively. This temperature increase is accurately represented within the framework of thermomechanical coupling. The temperature will continue to increase and reach saturation once adiabatic conditions are achieved, indicating that the test duration is insufficient to exchange the accumulated heat with the surrounding environment. The minor discrepancies observed can be attributed to the assumption of constant thermal properties, which in reality may influence the temperature field. Nevertheless, it is important to highlight that a straightforward SE-based simulation test can already offer very valuable insights into the thermomechanical response of a material at a singular point, facilitating very rapid evaluation of its performance.

## 5. Conclusions

This work outlines a comprehensive workflow for the thermomechanical characterization of pure polymers and their blends, showcasing state-of-the-art experimental and numerical techniques to develop high-performance polymer blends.

In this study, both pure polymers (PP and HDPE) and a PP/HDPE binary polymer blend were characterized using advanced experimental techniques. The integration of a 3D stereo digital image correlation (DIC) system with an Infrared (IR) camera revealed the self-heating effects on mechanical performance.

By isolating the intrinsic response of the material from the nonlinear geometric effects induced by the dog-bone specimen shape, the true stress–strain response was accurately characterized using an advanced polymer constitutive model. The predictions from this model closely matched the observed mechanical behavior and temperature evolution for all materials studied, demonstrating its flexibility in capturing varying stress–strain trends and underlying physical mechanisms. The rate-dependence observed in PP/HDPE blends closely resembled that of pure HDPE, despite the blends achieving higher stress levels due to the reinforcing presence of PP.

This methodology offers a unified approach for high-fidelity, rapid evaluation, enhancing the potential for broader industrial applications of future high-performance polymer blends.

## Figures and Tables

**Table 1:** Injection molding parameters.

Set Parameters	Unit	Value
Injection speed	mm/s	150
Injection pressure	bar	152
Holding pressure	bar	103
Holding time	s	3.5
Cycle time	s	48

**Table 2:** Set of material parameters required for the USCP model.

Material Parameter	Unit	Description	HDPE	PP	PP/HDPE
ρ	kg·m^−3^	Density	945	905	941 *
Resistance I (Amorphous)					
$E_{a,\mathrm{ref}}$	MPa	Modulus at _*θ*ref_	394	1000	590
_*θ*ref_	K	Reference temperature	302.15	304.15	304.15
β	1/K	Temperature dependence	0.019	0.001	0.02
$\nu_{I}$	-	Poisson’s ratio	0.45	0.43	0.45
$s_{0}$	MPa	Initial equivalent strength	16.73	39.28	24.38
$s_{1}$	MPa	Athermal peak strength	25.02	45.48	33.05
$s_{2}$	MPa	First saturation strength	25.5	49	34.01
$h_{1}$	MPa	Pre-peak hardening	325	536	500
$h_{2}$	MPa	Post-peak softening	405	367	403
${\bar{\varepsilon }}_{p}$	-	Peak plastic strain	0.08696	0.17584	0.0882
*f*	-	Smooth factor	0.3	0.3	0.3
α	-	Pressure sensitivity	0	0	0
*m*	-	Rate sensitivity	0.66	0.66	0.66
${\bar{\varepsilon }}_{0}$	1/s	Rate sensitivity	0.0208	0.395	0.546
*A*	K/MPa	Rate sensitivity	270.5	164.8	202.4
Resistance I (Crystalline)					
${\bar{\varepsilon }}_{c}$	-	Activation plastic strain	0.197	0.5	0.15
$s_{3}$	MPa	Second saturation strength	29	33	40
$h_{3}$	MPa	Second yield hardening	160	10020	120
Resistance N (Rubber effect)					
$C^{R}$	MPa	Rubbery modulus	1.8	6	1.6
*N*	-	Number of rigid links	4	225	4
Thermal properties					
*k*	W·m^−1^·K^−1^	Thermal conductivity	0.51	0.27	0.48 *
$c_{p}$	J·kg^−1^·K	Specific heat	2900	1570	2767 *

* Calculated using Equations (9) and (10).

## Data Availability

Data are contained within the article.
